# Oxygen Vacancy Injection on (111) CeO_2_ Nanocrystal Facets for Efficient H_2_O_2_ Detection

**DOI:** 10.3390/bios12080592

**Published:** 2022-08-03

**Authors:** Tong Li, Qi Wang, Zhou Wang

**Affiliations:** Key Laboratory of Liquid-Solid Structural Evolution and Processing of Materials of Ministry of Education, School of Materials Science and Engineering, Shandong University, Jinan 250061, China; litong1222@hotmail.com (T.L.); wangqi1016@sdu.edu.cn (Q.W.)

**Keywords:** electrochemical H_2_O_2_ sensing, oxygen vacancy, facet, cerium dioxide

## Abstract

Facet and defect engineering have achieved great success in improving the catalytic performance of CeO_2_, but the inconsistent reports on the synergistic effect of facet and oxygen vacancy and the lack of investigation on the heavily doped oxygen vacancy keeps it an attractive subject. Inspired by this, CeO_2_ nanocrystals with selectively exposed crystalline facets (octahedron, cube, sphere, rod) and abundant oxygen vacancies have been synthesized to investigate the synergistic effect of facet and heavily doped oxygen vacancy. The contrasting electrochemical behavior displayed by diverse reduced CeO_2_ nanocrystals verifies that oxygen vacancy acts distinctly on different facets. The thermodynamically most stable CeO_2_ octahedron enclosed by heavily doped (111) facets surprisingly exhibited the optimum non-enzymatic H_2_O_2_ sensing performance, with a high sensitivity (128.83 µA mM^−1^ cm^−2^), a broad linear range (20 µM~13.61 mM), and a low detection limit (1.63 µM). Meanwhile, the sensor presented satisfying selectivity, repeatability, stability, as well as its feasibility in medical disinfectants. Furthermore, the synergistic effect of facet and oxygen vacancy was clarified by the inclined distribution states of oxygen vacancy and the electronic transmission property. This work enlightens prospective research on the synergistic effect of alternative crystal surface engineering strategies.

## 1. Introduction

Hydrogen peroxide (H_2_O_2_) is a common oxidizing agent and an essential intermediate in biomedical, pharmaceutical, industrial, and environmental fields, as well as enzymatic reactions [1,2,3,4,5]. Therefore, it is of great significance to develop efficient techniques for the reliable, accurate, and fast detection of H_2_O_2_. Although enzymatic H_2_O_2_ sensing electrodes are widely used to provide the desired specificity and sensitivity, the sophisticated immobilization processes, instability, and high cost impede their further popularization. Fortunately, the emergence of inorganic nanocatalysts blazes a new trial in the application of non-enzymatic electrochemical sensors due to their excellent enzyme-mimetic catalytic properties, high stability, and low budget [6,7,8,9]. Among the various inorganic catalysts, CeO_2_ has been extensively used as an active component due to its outstanding redox property, controlled morphology, abundant reserves, and non-toxic nature [10,11,12,13].

Exposed facets and oxygen vacancies (denoted as OVs hereafter) display crucial roles in achieving superior catalytic activity. Atomic arrangement, electron distribution, coordination state, and surface energy on different facets differs, which endows nanocrystals with diverse catalytic activity [14,15,16]. OVs have also been confirmed beneficial in accelerating electron transfer, facilitating adsorption of reactant, lowering barrier energy of reactions, and increasing catalytic active sites [17,18,19]. Inspired by the positive effect reported, the role of facets and OVs in CeO_2_ has been extensively studied. Datye and Henson explored that Pd supported on (111) surfaces of ceria rods exhibits room-temperature CO oxidation activity, while Pd on (100) surface of ceria cubes shows comparable activity at a temperature higher than 60 °C [20]. Zhang and Shi reported that Cu doping can increase the O vacancy concentration in CeO_2–*x*_ and promote the photocatalytic activity of CeO_2−*x*_ by ~26 times [21]. Jiang et al. proposed that CeO_2_ nanorods with well-defined {110} and {100} crystal facets exhibits superb catalytic activity H_2_S selective oxidation due to the higher concentration of surface oxygen vacancies [22]. Zhan et al. also found that a Ru/CeO_2_ catalyst possesses more oxygen vacancy when a CeO_2_ rod with a predominantly exposed {110} surface facet is used, which provides higher ability in propane combustion [23]. Peng and Si reported the roles of different OVs in toluene combustion, which unraveled that surface vacancies tend to adsorb and activate gaseous O_2_ to form adsorbed oxygen species, whereas bulk vacancies improve the mobility and activity of lattice oxygen species [24]. Although great successes have been achieved in studying the multiple features of facets and OVs in CeO_2_, the relationship between facets and OVs, especially heavy-doped OVs, is still unclear since the majority of previous studies focused on the spontaneously generated OVs with low concentration and random distribution.

In 2005, Esch et al. discovered the unraveled oxygen vacancy clusters in CeO_2_ via high-resolution STM and found the cluster play crucial roles in the redox properties of CeO_2_ catalysts [25]. Li et al. further proved that the vacancy clusters were more active than the mono-dispersed vacancies in CO oxidation [26]. According to previous literature, OVs tend to cluster on reduced CeO_2_ (111) planes and preferentially align in the <111> direction, which has been proposed in theoretical explorations [27] and proved by observations through high-resolution STM [25], atom-resolved noncontact atomic force microscopy [28], and dynamic force microscopy [29]. However, OVs in an octahedral CeO_2_ nanocrystal with a (111) surface always failed to provide superior performance [22,23,24], which makes it an interesting research subject to discover the technique of efficiently introducing sufficient OVs into CeO_2_ (111) and to verify the synergistic interactions between heavily doped OVs and facets.

In this work, CeO_2_ octahedrons with selectively exposed (111) facets were synthesized and subsequently introduced with abundant OVs through aluminothermic reduction. Systematic characterizations, including morphology, phase, electron structure, and H_2_O_2_ sensing performance, were carried out on CeO_2_ octahedrons before/after reduction to determine the effect of injected OVs on a (111) facet. In order to verify the synergistic effect of OVs and facets, the comparative electrocatalytic investigation was conducted on CeO_2_ nanocrystals with diverse exposed facets before and after reduction. Furthermore, the mechanism of the extraordinary effect of OVs in CeO_2_ octahedron was elaborated from the perspective of the distribution states of OVs and the electron-transfer characteristic. Our work provides a new idea to developing high-efficiency catalysts and might enlighten the prospective research regarding the synergistic effect of different crystal surface engineering strategies.

## 2. Materials and Methods

### 2.1. Reagents and Apparatus

All reagents were purchased from suppliers and were used directly without purification. Cerium nitrate hexahydrate (Ce(NO_3_)_3_.6H_2_O, 99.95%), sodium phosphate tribasic dodecahydrate (Na_3_PO_4_·12H_2_O, 98.0%, AR), D-(+)-galactose (C_6_H_12_O_6_, 99%), D-Fructose (C_6_H_12_O_6_, 99%), ascorbic acid (AA, C_6_H_8_O_6_, 99.99%), uric acid (UA, C_5_H_4_N_4_O_3_, 99%), and dopamine hydrochloride (DA, C_8_H_11_NO_2_·HCl, 98%) were purchased from Aladdin Reagent. Sodium hydroxide (NaOH, ≥98%), ethylene glycol (C_2_H_6_O_2_, >99.5%, AR), aluminum powder (Al, 100–200 mesh, 99%), acetone (C_3_H_6_O, ≥99.5%, AR), iodine (I_2_, ≥99.8%, AR), sodium dihydrogen phosphate dihydrate (NaH_2_PO_4_·2H_2_O, >99.5%, AR), disodium hydrogen phosphate dodecahydrate (Na_2_HPO_4_·12H_2_O, ≥99.0%, AR), sodium chloride (NaCl, ≥99.5%, AR), and ethanol absolute (C_2_H_6_O, ≥99.7%, AR) were purchased from Sinopharm Chemical Reagent. Polyvinylpyrrolidone (PVP, MW = 40000) was purchased from VETEC Reagent. Potassium chloride (KCl, ≥99.5%) and hydrogen peroxide 30% (H_2_O_2_, 30%, GR) were purchased from Tianjin Kermel Chemical Reagent. α-D-glucose (C_6_H_12_O_6_, 96%) was purchased from Sigma Aldrich Reagent. Fluorine-doped Tin Oxide (FTO) was purchased from Zhuhai Kaivo Optoelectronic Technology Co., Ltd. (Zhuhai, China). The real medical disinfectant, mainly composed of sodium hypochlorite, surfactants, NaOH, and compound essences was purchased in the supermarket.

X-ray diffraction patterns (XRD) were recorded by a Rigaku DMAX-2500PC diffractometer with Cu Kα radiation (λ = 0.15406 nm) (scanning rate: 10°/min). Field emission scanning electron micrograph (FE-SEM) was acquired on a JEOL JEM-7800F. Transmission Electron Microscopy (TEM) images were taken with JOEL JEM-2100F operated at 200 kV. Electron paramagnetic resonance (EPR) test was carried out on a JES-X320 electron spin resonance spectrometer at room temperature. X-ray photoelectron spectroscopy were analyzed by a Kratos AXIS Supra using Mg Kα radiation of 1253.6 eV. The XPS spectra were charge corrected with the C 1s peak at 284.8 eV.

### 2.2. Preparation of CeO_2_ with Selectively Exposed Facet

**Scheme 1:**
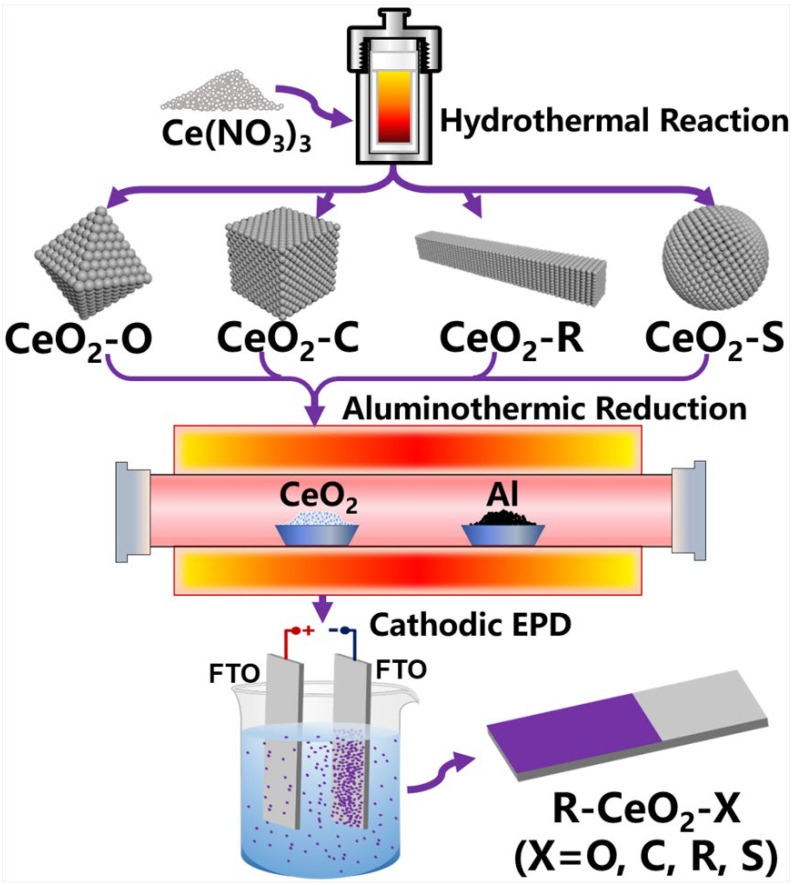
Schematic diagram of the fabrication procedures.

The fabrication procedures, including hydrothermal reaction, aluminothermic reduction, and electrophoretic deposition (EPD), are outlined in Scheme 1.

#### 2.2.1. Preparation of CeO_2_ with Selectively Exposed Facet

CeO_2_ octahedron: Firstly, 0.08 mmol Na_3_PO_4_·12H_2_O was dissolve in 64 mL deionized water. After complete dissolution, 8 mmol Ce(NO_3_)_3_·6H_2_O was added. The solution was kept under magnetic stirring for 1 h at room temperature. Then the solution was transferred into an 80 mL Teflon-lined stainless-steel autoclave and heated at 160 °C for 12 h. After hydrothermal reaction, the powder at the bottom of the Teflon-lined autoclave was centrifuged and washed with deionized water and ethanol until pH = 7. Finally, the samples were dried at 60 °C for 24 h and then calcined at 600 °C for 5 h at a ramp rate of 5 °C/min in a muffle furnace. The obtained CeO_2_ octahedral samples are marked as CeO_2_-O.

CeO_2_ cube and rod: Firstly, 3.2 mmol Ce(NO_3_)_3_·6H_2_O and 0.384 mol NaOH were dissolved in 64 mL deionized water under magnetic stirring for 30 min. Then the solution was transferred to an 80 mL Teflon-lined stainless steel autoclave and heated for 24 h (cube: at 180 °C, rod: at 100 °C). After hydrothermal reaction, the powder was centrifuged and washed with deionized water and ethanol until pH = 7. Finally, the samples were dried at 60 °C for 24 h and then calcined at 600 °C for 5 h at a ramp rate of 5 °C/min in a muffle furnace. The obtained CeO_2_ cube and rod samples are marked as CeO_2_-C and CeO_2_-R, respectively.

CeO_2_ sphere: Firstly, 4.6 mmol Ce(NO_3_)_3_·6H_2_O and 0.8 g PVP were dissolved in 56 mL ethylene glycol, then 8 mL deionized water was added into the solution under magnetic stirring for 30 min. Then the solution was transferred to an 80 mL Teflon-lined stainless steel autoclave and heated at 160 °C for 24 h. After hydrothermal reaction, the powder was centrifuged and washed with deionized water and ethanol until pH = 7. Finally, dried at 60 °C for 24 h, calcined at 600 °C for 5 h at a rate of 5 °C/min in a muffle furnace. The obtain CeO_2_ sphere samples are marked as CeO_2_-S.

#### 2.2.2. Non-Contacting Reduction Process of CeO_2_

Rapid aluminothermic reaction is an efficient technology recently applied to reduce various oxides [30] and was adopted to introduce abundant OVs into CeO_2_. An alumina calcination boat, on the bottom of which the prepared CeO_2_ samples were evenly spread, was placed at the far-end of the quartz tube. Another calcination boat containing excess aluminum powder was placed at the near-end of the pump. The tube was pre-evacuated to a pressure of less than 6 × 10^−4^ Pa. Then the CeO_2_ samples and aluminum powder were both heated to 700 °C for 3 h at a heating rate of 5 °C/min. After cooling to room temperature, the reduced CeO_2_ samples were obtained and denoted as R-CeO_2_-X (X = O, C, R or S).

#### 2.2.3. Preparation of Electrode

Before the formal filming process, the FTO substrates were cleaned with following the previous reports [31,32,33]. CeO_2_-assembled FTO electrodes were prepared through electrophoretic deposition. CeO_2_ powder (9.6 mg) and iodine (2.4 mg) were dispersed in acetone (20 mL) by ultrasonic treatment. The reaction of acetone and iodine produces free protons that will adsorb on the CeO_2_ particles in the suspension and increase their surface charges, thus facilitating the electrophoretic process. Two pieces of FTO were placed parallel and held in the above solution for 5 min under a bias voltage of 10 V. The deposition area was fixed at 1 cm^2^ by controlling the immersion area during the electrophoretic process. Finally, the uniformly deposited FTO on the cathode was dried in a vacuum oven at 150 °C for 2 h to remove iodine.

### 2.3. Electrochemical Measurements

All electrochemical measurements were performed on an Ivium Vertex One EIS electrochemical workstation. The electrochemical tests were carried out in a conventional three-electrode system with a 1.5 cm × 1.5 cm Pt plate as counter electrode, an Ag/AgCl electrode as reference electrode, CeO_2_-assembled FTO as working electrode, and 0.1 M PBS as electrolyte. The electrolyte for the electrochemical measurements was a N_2_-saturated 0.1 M phosphate buffer (PBS, pH = 7.4) in the absence/presence of H_2_O_2_.

## 3. Results and Discussion

### 3.1. Characterization of OVs in CeO_2_-O

**Figure 1:**
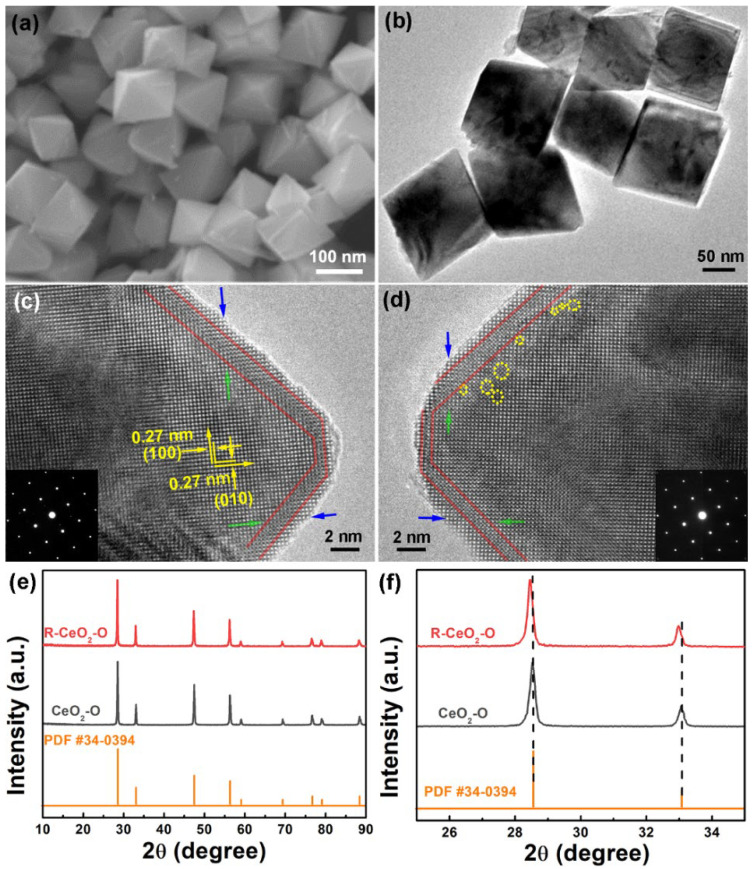
(**a**) SEM, (**b**) TEM, and (**c**) HRTEM images of CeO_2_-O. (**d**) HRTEM image of R-CeO_2_-O. (**e**,**f**) XRD patterns of CeO_2_-O and R- CeO_2_-O.

The effect of OVs on the morphology of CeO_2_ nanocrystals was investigated by comparable studies on CeO_2_-O and R-CeO_2_-O. As shown in the scanning electron microscope (SEM) and transmission electron microscopy (TEM) images (Figure 1a,b), the monodispersed nanocrystals (CeO_2_-O), with a side length of about 150 nm, were successfully fabricated. After aluminothermic reduction, the octahedral morphology is well-preserved (Figure S1). In the view of high-resolution transmission electron microscopy (HRTEM) (Figure 1c,d), the lattice fringe of both samples almost reaches the edge of crystals, indicating high crystallinity across the whole grain. According to Figure 1c, CeO_2_-O presents a determined interplanar spacing of 0.27 nm, which corresponds to the {100} crystal plane group. Since the electron beam of TEM was irradiated on the octahedron along the 〈001〉 direction, the exposed facets of CeO_2_-O can be indexed as {111} crystal plane group through simple crystallographic calculation. The inset SAED patterns reveal clear diffraction spots, indicating the monocrystalline structure for both samples. In Figure 1d, no trace of surface disorder layer, which was usually detected in thermally reduced nanomaterials in previous reports [30,34], can be verified in R-CeO_2_-O. However, the close observation of atom configuration discovers the imperfect periodicity of the lattice. On the one hand, both samples show a transition layer, with a thickness of 4–6 atomic layers, between the surface layer (3–5 atomic layers) and bulk, as separated with red lines. Lattice fringes are cut off by the transition layer, presenting numerous mismatched atomic layers (marked with green or blue arrows) as a typical Shockley stacking fault. On the other hand, some additional indistinct and uneven areas (marked with yellow dotted circles) can be verified according to the contrast difference in Figure 1d, confirming the higher concentration of OVs in R-CeO_2_-O.

Besides direct observation through high-resolution imaging instruments like TEM, STEM, AFM, etc., the defects can also be indirectly detected through multiple conventional strategies. Firstly, the lattice stretching induced by OVs can be monitored by XRD patterns of R-CeO_2_-O and CeO_2_-O, as shown in Figure 1e. Both samples show identical diffraction patterns with nine characteristic peaks at 2 θ = 28.6°, 33.1°, 47.5°, 56.3°, 59.1°, 69.4°, 76.7°, 79.1°, and 88.4°, corresponding to (111), (200), (220), (311), (222), (400), (331), (420), and (422) facets of face-centered cubic (FCC) fluorite structure with space group Fm-3m of CeO_2_ crystal (PDF #34-0394), respectively. In order to get a clear view, the regions with the diffraction angle of 25°–35° are magnified and shown in Figure 1f. The slight shift of characteristic peaks towards lower angle direction for R-CeO_2_-O implies an expansion of lattice after aluminothermic reduction reaction, which can be ascribed to the repulsive force of charged OVs [25,26,27,28,29].

**Figure 2:**
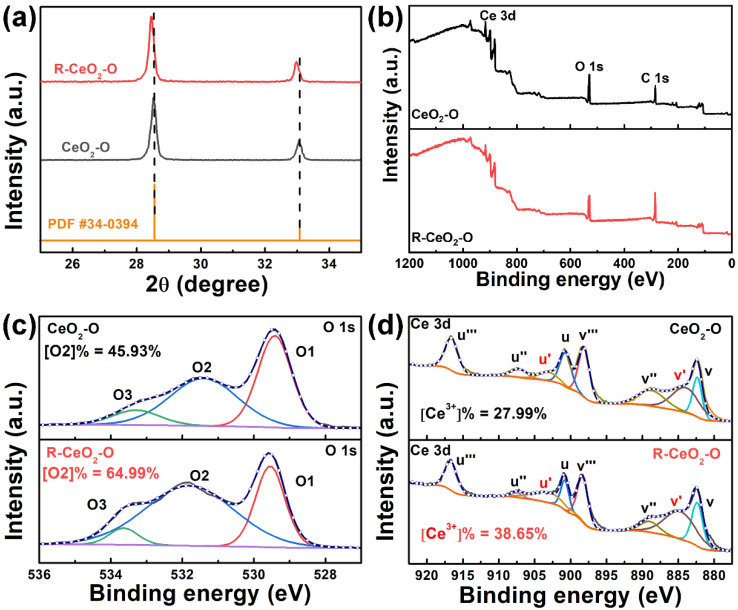
(**a**) Raman, XPS (**b**) survey, (**c**) O 1s, and (**d**) Ce 3d spectra of CeO_2_-O and R-CeO_2_-O.

Secondly, according to previous report [24], the concentration of OVs in CeO_2_ samples can be evaluated by a Raman test, which was conducted on CeO_2_-O and R-CeO_2_-O and the normalized spectra were recorded in Figure 2a. Four obvious peaks can be observed in both curve, which are located at 257 cm^−1^, 462 cm^−1^, 600 cm^−1^, and 1170 cm^−1^. Among them, the distinct characteristic peak at 462 cm^−1^ belongs to the F_2g_ mode in fluorite structure CeO_2_ [35], which is attributed to a symmetrical stretching mode of the vibrating unit between Ce^4+^ and the surrounding eight oxygen atoms [36,37,38]. The weak characteristic peak at 257 cm^−1^, 600 cm^−1^, and 1170 cm^−1^ is attributed to the second-order transverse acoustic (2TA), defect-induced (D), and longitudinal optical (2LO) modes [22]. The presence of OVs in CeO_2_ crystal can be reflected in the peak intensity of D mode [36,39,40]. Generally, the ratio of the intensity of the strong characteristic peak at 600 cm^−1^ to the weak characteristic peak at 462 cm^−1^ (I_600_/I_462_) can be applied to evaluate the concentration of OVs [24]. In Figure 2a, R-CeO_2_-O shows obviously higher intensity at D mode peak. Since the peak intensity at 600 cm^−1^ has been normalized, R-CeO_2_-O obtains much higher ratio of I_600_/I_462_, indicating increased OVs in CeO_2_-O. The relative concentration of OVs was further confirmed by calculating the nature of the major peak at 462 cm^−1^ based on the spatial correlation model [37,41]. The relative values are 6.3 × 10^21^ cm^−3^ (CeO_2_-O) and 13.3 × 10^21^ cm^−3^ (R-CeO_2_-O), respectively, which is consistent with the result of I_600_/I_462_.

Thirdly, the residual electrons from the extracted oxygen atoms are commonly believed to transfer to the neighboring Ce atoms and partially fill Ce *f* orbit [25,29,42], inducing modifications of magnetic properties. Electron paramagnetic resonance (EPR) has been widely applied to semi-quantitatively analyze the OVs in transition metal oxide [30,34]. As shown in Figure S2, the recorded spectra of CeO_2_-O and R-CeO_2_-O both have a fingerprint signal at g = 1.997, which originates from the unpaired electrons on the *f* orbit of Ce^3+^ [43]. The obviously stronger signal for R-CeO_2_-O suggests that abundant OVs and Ce^3+^ are generated in R-CeO_2_-O.

Lastly, the electron transportation from OVs to neighboring Ce atoms naturally changes the electronic structure, which brings X-ray photoelectron spectroscopy (XPS) an effective approach to investigate OVs. Figure 2b shows the wide XPS survey spectra of CeO_2_-O and R-CeO_2_-O, which confirm the identical pure composition of Ce and O elements. The O 1s spectra shown in Figure 2c can be deconvoluted into three individual peaks, which are denoted as O1, O2, and O3, respectively [44]. The O1 peak at 529.5 eV represents the metal–oxygen bond (Ce-O bond) and the O2 peak at 531.4 eV is attributed to the OVs, while the O3 peak at 533.3 eV can be ascribed to the oxygen in the adsorbed hydroxyl group on the surface [45]. The concentration of OVs can be determined through dividing the total peak area of (O1 + O2 + O3) by the peak area of O2, which reveals that the proportion of O2 in R-CeO_2_-O (64.99%) is higher than that in CeO_2_-O (45.93%). The higher concentration of O2 indicates that extra OVs are created in R-CeO_2_-O. The spectra of Ce 3d can be deconvoluted into two groups of partially overlapping peaks. The peaks corresponding to Ce 3d_3/2_ are labeled with group u (u-u’’’), while peaks related with Ce 3d_5/2_ are labeled with group v (v-v’’’). All peaks are specifically expressed as u’’’ (916.6 eV), u‘‘ (907.2 eV), u‘‘ (903.1 eV), u (900.7 eV), v’’ (898.1 eV), v’’ (888.7 eV), v’’ (884.0 eV), and v (882.4 eV), where u’ and v’ correspond to Ce^3+^ and the remaining six peaks correspond to Ce^4+^ (Figure 2d) [46]. The content of Ce^3+^ can be estimated by the peak area ratio of Ce^3+^ (u’ and v’) to all the fitted peaks, which is calculated to be 27.99% for CeO_2_-O and 38.65% for R-CeO_2_-O, respectively. The superior concentration of Ce^3+^ in R-CeO_2_-O, inconsistent with its higher proportion of O2, verifies the efficient injection of OVs, which is of great significance and may contribute to improving catalytic performance.

Above all, abundant additional OVs and Ce^3+^, which will play an important role in improving H_2_O_2_ electrochemical performance, are efficiently introduced into R-CeO_2_-O by aluminothermic reaction, as proved by multiple characterizations.

### 3.2. Effect of OVs on H_2_O_2_ Sensing Performance

**Figure 3:**
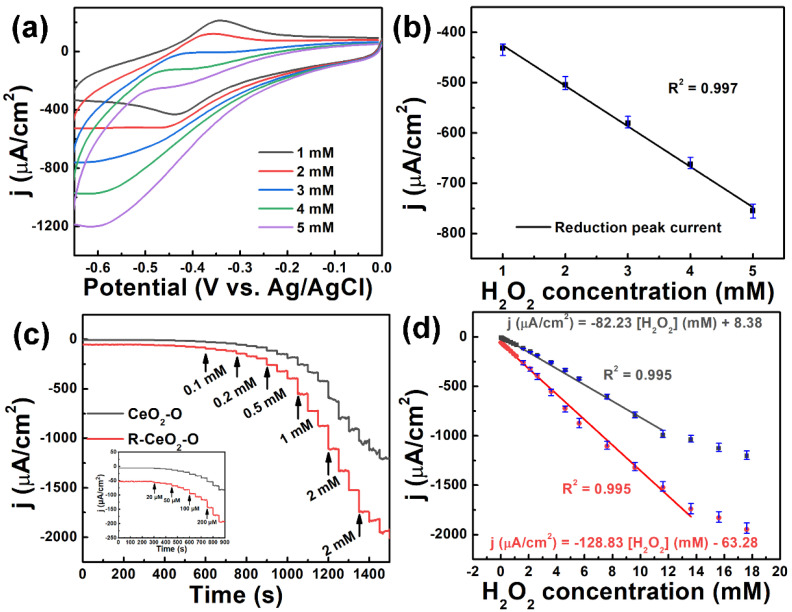
(**a**) CV scans of R-CeO_2_-O in 0.1 M PBS with different concentration of H_2_O_2_ and (**b**) linear plot of cathodic peak current density versus H_2_O_2_ concentrations. (**c**) Amperometric response of R-CeO_2_-O after continuous injection of H_2_O_2_ in 0.1 M PBS solution with a bias of −0.5 V. (**d**) Linear plots of current density versus H_2_O_2_ concentrations.

In order to present a comprehensive and precise view of the electrochemical behavior of R-CeO_2_-O, systematic electrochemical tests were carried out in a three-electrode system. At first, CV tests were conducted in electrolytes containing different amounts of H_2_O_2_ in order to preliminarily evaluate the linearity of current response, which is a key index for sensors. As shown in Figure 3a, the cathodic peak currents of the CV curves gradually increase with the elevating concentration of H_2_O_2_ (1 mM–5 mM). Meanwhile, the peak position gradually shifts to the negative direction, which is attributed to the restricted diffusion process of H_2_O_2_. R-CeO_2_-O shows an excellent linear relationship between cathodic peak current density and the concentration of H_2_O_2_ in Figure 3b, which indicates its potential to achieve superior H_2_O_2_ catalytic performance.

Further evaluation of the sensing performance relies on the precise measurement of key indexes, such as sensitivity, linear range, detection limit, selectivity, stability, and reproducibility, which were all investigated via chronoamperometry (CA) method in this work. Before the formal CA test, the optimal operation voltage of chronoamperometry test was determined by recording the Current density vs. Time curves with continuous injection of H_2_O_2_ (1 mM) into 0.1 M PBS per 50s under different bias voltages (−0.3~0.6 V). As shown in Figure S3, the current responds as soon as the H_2_O_2_ is injected and quickly reaches a steady state, which indicates that H_2_O_2_ can be rapidly adsorbed, activated, and decomposed on the surface of R-CeO_2_-O. The current density elevates with the increasing bias voltage until the voltage reaches −0.6 V, at which the amperometric curve can hardly be distinguished from the curve recorded at −0.5 V. In order to prevent excessive negative potential and possible interference [47], a bias of −0.5 V was applied as the operation potential for further CA tests. As presented by Figure 3c, the amperometric response of CeO_2_-O and R-CeO_2_-O both increases with continuous injection of H_2_O_2_, while R-CeO_2_-O obtains significantly higher current density than CeO_2_-O, testifying the boosted activity of R-CeO_2_-O in catalyzing H_2_O_2_ reduction. The calibration curve shown in Figure 3d evidences the well-fitted linear relationship between H_2_O_2_ concentration and current density. The sensitivity of R-CeO_2_-O is calculated to be 128.83 μA·mM^−1^·cm^−2^ with a wide linear detection range of 20 μM–13.61 mM and a low detection limit of 1.63 μM (S/N = 3) (linear regression equation: j (μA/cm^2^) = −128.83 [H_2_O_2_] (mM) − 63.28 (R^2^ = 0.995)). For comparison, CeO_2_-O presents a decaying sensitivity (82.23 μA·mM^−1^·cm^−2^) with narrowed linear detection range (20 μM–1.61 mM) and higher detection limit of 2.45 μM (S/N = 3) (linear regression equation: j(μA/cm^2^) = −82.23 [H_2_O_2_] (mM) + 8.38 (R^2^ = 0.995)).

In order to obtain an objective evaluation of CeO_2_-O and R-CeO_2_-O sensors in our work, their typical sensing-performance indicators were compared with other CeO_2_-containing H_2_O_2_ sensors available in previous reports [38,48,49,50,51,52,53]. The collected data shown in Table 1 verifies the leading position of R-CeO_2_-O and CeO_2_-O sensor with impressive sensitivity and wide linear range among recent CeO_2_-dominated H_2_O_2_ sensors [48,49,50]. Furthermore, the R-CeO_2_-O and CeO_2_-O sensors show comparable (even better) performance to some CeO_2_-containing sensors assembled with other electro-active catalysts such as noble metal [38,49,51,52] and conductive nanocarbon species [38,53]. Thus, the synergistic design of crystal facet and defect dependent nano-ceria, realizes the superior H_2_O_2_ electrochemical sensing performance, providing a facile and cost-effective approach in developing high-quality non-enzymatic electrochemical sensors.

### 3.3. Synergistic Effect of Facets and Oxygen Vacancies

In order to investigate the comprehensive effect of facet and oxygen vacancy on H_2_O_2_ sensing performance, CeO_2_ nanocrystals with other different exposed facets were successfully fabricated and their morphologies and phase were characterized by SEM and XRD (Figure S4). According to the previous reports, the surface of CeO_2_-C, CeO_2_-R, and CeO_2_-S are dominated by (100), (110) + (100), (111 + 100) facets, respectively [22,54,55,56]. The N_2_ adsorption–desorption isotherms test was also conducted, as shown in Figure S5a. All samples exhibit the H3 type hysteresis loop due to the accumulation of nanoparticles. The specific surface area was calculated and ranks in the order of R-CeO_2_-S (117.5 cm^3^/g) > R-CeO_2_-R (57.2 cm^3^/g) > R-CeO_2_-C (21.7 cm^3^/g) > R-CeO_2_-O (10.6 cm^3^/g). The Raman spectra of reduced CeO_2_ nanocrystals (Figure S5b) were recorded to determine the concentration of OVs, which was calculated to be 13.3 × 10^21^ cm^−3^ (R-CeO_2_-O), 20.3 × 10^21^ cm^−3^ (R-CeO_2_-C), 15.9 × 10^21^ cm^−3^ (R-CeO_2_-R), and 28.5 × 10^21^ cm^−3^ (R-CeO_2_-S) respectively.

**Figure 4:**
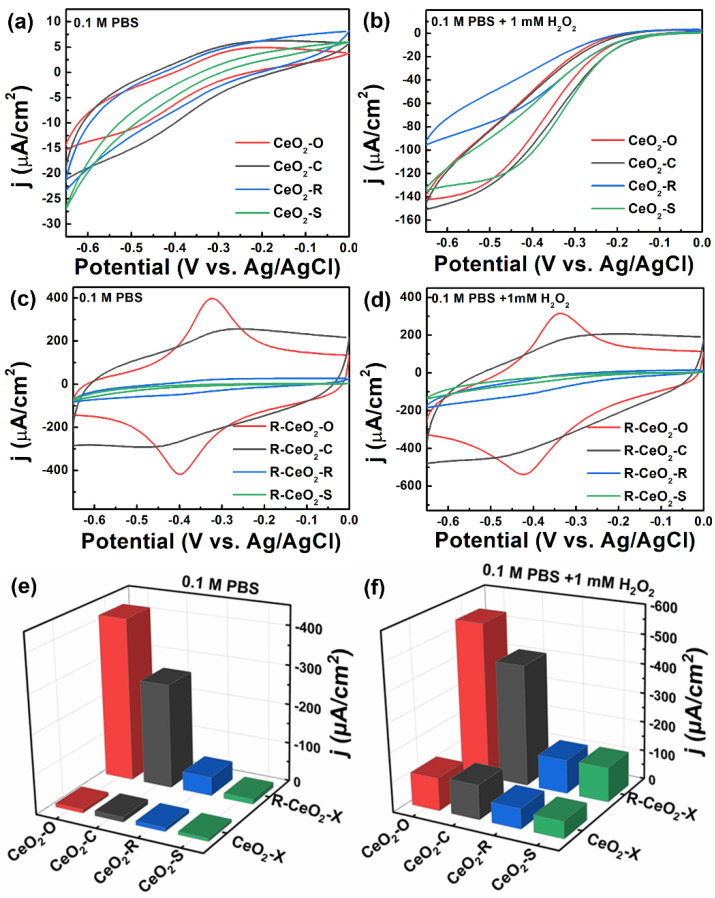
CV curves of CeO_2_-X samples in N_2_-saturated 0.1 M PBS containing (**a**) 0 mM and (**b**) 1 mM H_2_O_2_; CV curves of R-CeO_2_-X samples in N_2_-saturated 0.1 M PBS containing (**c**) 0 mM and (**d**) 1 mM H_2_O_2_. 3D histograms of reduction peak currents for CeO_2_ with four different morphologies before and after reduction in N_2_-saturated 0.1 M PBS containing (**e**) 0 mM and (**f**) 1 mM H_2_O_2_.

In order to explore the synergistic effect of facets and OVs, the electrochemical performance of CeO_2_ samples with different morphologies before/after reduction was studied by cyclic voltammetry (CV) test in N_2_-saturated PBS solutions in the absence/presence of 1 mM H_2_O_2_ at the scan rate of 50 mV/s. Specifically, in Figure 4a, the CV curves of all pristine samples show low current density and identical oval shape with no shape redox peaks. After adding 1 mM H_2_O_2_, the reduction current density of all pristine samples increases slightly; however, the CV curves turn more oblique in shape (Figure 4b), indicating low catalytic activity and poor electrochemical kinetics. After aluminothermic reduction, the current response of all samples increases significantly (Figure 4c), which can be ascribed to the injection of abundant Ce^3+^ and OVs. Generally speaking, the removal of oxygen atoms from lattice improves electrochemical catalytic performance in two aspects. Firstly, two electrons previously bound to the discharged oxygen remain in the oxide and populate defect states located inside the bandgap (in-gap states). When these donor states are shallow, some electrons can delocalize into the conduction band, where they can conduct electrons in a nominally insulating material. Therefore, the conductivity of n-type oxides can be improved by introducing OVs into the lattice, which is also observed in SrTiO_3_ [57]. Secondly, the discharge of oxygen atoms leaves abundant unsaturated dangling bonds on the surface or sub-surface, hence facilitating the adsorption of reactants. Meanwhile, the charge accumulation regions around OVs, induced by localized electrons, can activate the adsorbed species and decrease the reaction energy barrier. Consequently, OVs are capable of acting as active reaction centers.

Based on the mechanisms above, the current density should show a positive correlation with the concentration of OVs. However, R-CeO_2_-O enclosed by (111) planes are thermodynamically most stable and less prone to accommodate OVs, as can be seen from the order of vacancy formation energy on the low index surfaces ((111) > (100) > (110)) [58,59,60], obtain boosted current density, which is comparable with R-CeO_2_-C and much higher than R-CeO_2_-R and R-CeO_2_-S. Besides the elevated current density, it is interesting that the CV curves of R-CeO_2_-O and R-CeO_2_-C are distinct in shape (Figure 4c,d). The CV curve of R-CeO_2_-C is quasi-rectangle in shape with a dimly visible oxidation peak at −0.2 V to −0.35 V and a reduction peak at −0.35 V to −0.5 V, which corresponds to the redox reaction between Ce^3+^ and Ce^4+^. In contrast, the CV curve of R-CeO_2_-O shows two noteworthy redox peaks centered in −0.32 V and −0.40 V. The narrow peaks of Ce^3+^ ↔ Ce^4+^ have high intensity which illustrates that the redox reaction is completed in a restricted potential region, and favors the application as a signal amplifier of sensor.

The diverse effect of OVs on different facets can be distinguished from the abnormally elevated current density and the distinct shape of CV curves of R-CeO_2_-O. Firstly, OVs prefer to be generated in the surface layer of CeO_2_ (110) and (100) planes, but in the subsurface layer of (111) planes, as widely validated by DFT+U (density functional theory corrected for on-site Coulomb interactions) calculations [61,62] and experimental methods [63]. Moreover, OVs in the surface layer, directly exposed to environment species, are much likely to be occupied by adsorbed molecules, thus, deactivated for the redox catalysis. However, OVs located at the adjacent subsurface layer can be protected partially from the access of external substances, thus, available for the role of catalytic active center [42]. Therefore, the superior electrochemical performance of R-CeO_2_-O enclosed by (111) planes can be ascribed to the preferentially generated and well-preserved OVs-sub. Secondly, the interesting distinction in the shape of CV curves for R-CeO_2_-O and R-CeO_2_-C can be interpreted from the distribution of OVs, which has a direct impact on the electronic structure of the lattice around the OVs. As discussed in the Introduction, OVs tend to cluster on reduced CeO_2_ (111) planes and preferentially align in the <111> direction. Esch et al. found that almost all OVs (92% in the observation range) on CeO_2_ (111) planes induced by reduction are linear [25]. The high uniformity of the distribution of OVs on (111) will build identical electronic structures around all OVs, which contributes to the sharp redox peaks of the catalytic reaction. However, the distribution of OVs on CeO_2_ (100) is quite different. Owing to the close formation energy of OVs-sur and OVs-sub and the multiple distribution patterns of OVs, the electronic structure around each oxygen vacancy varies on CeO_2_ (100), which leads to the dispersive driving force (bias voltage) for the catalytic reaction and presents a CV curve with no characteristic peak.

After adding 1 mM H_2_O_2_ (Figure 4d), R-CeO_2_-O obtained the highest electrochemical response among four electrodes. The current density of the reduction peak for all samples were recorded as a histogram in Figure 4e,f. The proposed sensing reaction mechanism was shown as follows [64]:

Ce^4+^ + e^−^ → Ce^3+^ (1)

Ce^3+^ + H_2_O_2_ → Ce^4+^ + 2OH (2)

### 3.4. Mechanism of Boosted Sensing Performance on R-CeO_2_-O

**Figure 5:**
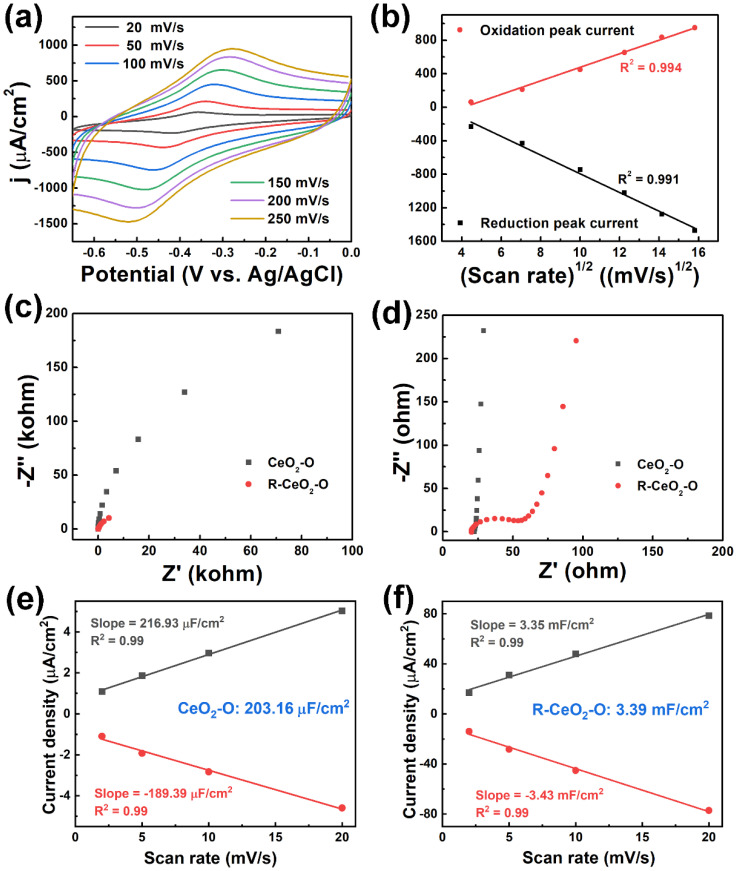
(**a**) CV scans of R-CeO_2_-O in 0.1 M PBS with 1 mM H_2_O_2_ at different scan rate and (**b**) linear plots of peak current density versus square root of scan rate. Nyquist plots of CeO_2_-O and R-CeO_2_-O (**c**) in full range and (**d**) in high-frequency region. The corresponding linear plot (derived from Figure S6) of the capacitive currents of (**e**) CeO_2_-O and (**f**) R-CeO_2_-O vs. scan rates for EASA calculation.

Firstly, the CV curves recorded at different scan rates was applied to study the reaction kinetics of R-CeO_2_-O, as shown in Figure 5a. With the increasing scan rate, the anodic peak of the CV curve gradually moves to the positive direction, while the cathodic peak shifts towards the negative direction. The good linear relation between the current density of the redox peak and the square root of the scan rate (Figure 5b) suggests that the H_2_O_2_ catalytic reduction on the surface of R-CeO_2_-O is a diffusion-controlled process, which means that the current response is closely related to the concentration of H_2_O_2_ and is limited by the diffusion of H_2_O_2_ in PBS.

Secondly, the electrochemical impedance spectroscopy (EIS) experiments were conducted to investigate the favorable effect of OVs on the electron transport characteristics of the electrodes. The obtained Nyquist plots are given in Figure 5c,d, in which the semicircle part in the high-frequency region represents the electron limited process. In the full range view (Figure 5c), a semicircle with an extremely large diameter was faintly displayed on the plot of CeO_2_-O, implying its huge charge transfer resistance (R_ct_), which is attributed to the insulating nature of pristine CeO_2_. The plot of R-CeO_2_-O can hardly be distinguished in Figure 5c. Therefore, the high-frequency part of the Nyquist plots were enlarged and a typical semicircle is discernible (Figure 5d), which verifies the dramatically decreasing R_ct_ for R-CeO_2_-O, suggesting that the high concentration of OVs in R-CeO_2_-O promote the electron transfer process. Moreover, judging from the intercept of the plots, which represents internal resistance (R_s_), R-CeO_2_-O also possesses relatively lower R_s_ than CeO_2_-O. The enhanced electron transport characteristics are ascribed to the delocalized electrons released from OVs by reduction.

Thirdly, the electrochemical active surface area (EASA) of CeO_2_-O and R-CeO_2_-O electrode was conducted on CeO_2_-O and R-CeO_2_-O electrode in N_2_-saturated 0.1 M PBS. The CV curves of CeO_2_-O and R-CeO_2_-O electrodes at different scanning rates in the non-faradic regions (−0.1 V–0 V) are shown in Figure S6. By taking the anode current density at −0.02 V and the cathode current density at −0.08 V, the slope was obtained after linear fitting (Figure 5e,f). Following the detailed method in the Supplementary Materials, EASA of the corresponding samples are 5.08 cm^2^ (CeO_2_-O) and 84.75 cm^2^ (R-CeO_2_-O), which verifies the obvious activation effect of OVs on (111) facet.

Given the above, the introduced OVs on the (111) surface of R-CeO_2_-O are capable of lowering the resistance and enhancing the concentration of active reaction sites, thus, achieving boosted H_2_O_2_ electrochemical sensing performance.

### 3.5. Investigation of Selectivity, Stability, Reproducibility and Practicability

**Figure 6:**
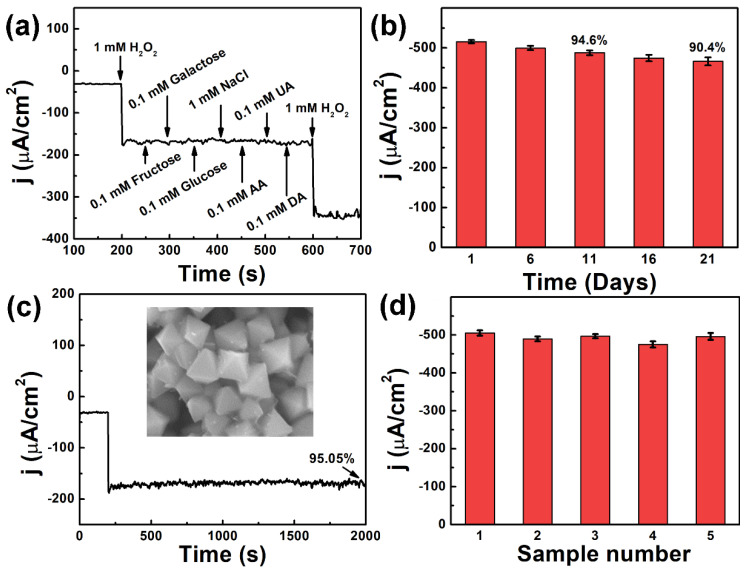
(**a**) Amperometric response of R-CeO_2_-O upon the successive addition of 1 mM H_2_O_2_, 0.1 mM fructose, 0.1 mM galactose, 0.1 mM glucose, 1 mM NaCl, 0.1 mM AA, 0.1 mM UA, 0.1 mM DA, and 1 mM H_2_O_2_ in 0.1 M PBS at a bias voltage of −0.5 V. (**b**) The current density response toward 1 mM H_2_O_2_ after different durations. (**c**) Response current density of R-CeO_2_-O during 2000 s upon addition of 1 mM H_2_O_2_. The inset in (**c**) shows the SEM image of R-CeO_2_-O after sensing. (**d**) Current density of five electrodes in response to 1 mM H_2_O_2_.

The selectivity, one of the most essential application indicators of a sensor, was evaluated by recording the current response to a group of common physiological-level electroactive substances, such as fructose, galactose, glucose, ascorbic acid (AA), uric acid (UA), dopamine (DA), and sodium chloride, by means of a CA test at the voltage of −0.5 V. As depicted in Figure 6a, after a regular step-wise amperometric response brought by the initial injection of 1 mM H_2_O_2_, the successive injection of interfering substances engenders no evident response, while a subsequent injection of 1 mM H_2_O_2_ prompts an identical increase to the first injection. Typical metal ions were also involved in the test, as shown in Figure S7. The superior current response towards the H_2_O_2_ injection in Figure 6a and Figure S7 proves the high selectivity of R-CeO_2_-O from two aspects. On the one hand, these disturbing substances cannot produce a comparable signal to H_2_O_2_; on the other hand, these disruptors are incapable of affecting the sensitivity of R-CeO_2_-O. The storage stability of R-CeO_2_-O electrodes was checked by storing them at room temperature (15–25 °C) in a general atmospheric environment and recording their current response in 0.1 M PBS with 1 mM H_2_O_2_ every five days. The histogram, shown as Figure 6b, verifies a retention of 90.4% of its initial amperometric response after 21 days, indicating its high storage stability. The stability in long-term consecutive operation was appraised by recording the CA curve after injecting 1 mM H_2_O_2_ to 0.1 M PBS (Figure 6c). The inject of H_2_O_2_ at 200 s induced a downward step on the current response, which keeps steady afterward and maintains 95.05% of the initial value after 30 min. Moreover, the octahedral morphology (inset of Figure 6c) is well preserved after the 2000 s CA test, corroborating the satisfactory stability. The investigation of reproducibility was carried out on five electrodes prepared independently following the same procedure. The amperometric response of five electrodes was measured in the presence of 1 mM H_2_O_2_, and a satisfied relative standard deviation (RSD) of 2.85% was achieved (Figure 6d). In summary, the above CA tests demonstrate that the R-CeO_2_-O sensor possesses good selectivity, high stability, and excellent reproducibility.

Furthermore, the sensing performance of our R-CeO_2_-O electrode in real medical disinfectant was investigated to find its feasibility in practical applications. Since H_2_O_2_ plays the role of sterilization, which can dominate the performance of disinfectant and guarantee medical safety, the content of H_2_O_2_ in medical disinfectant should be strictly monitored. After H_2_O_2_ solutions with known concentration was spiked to diluted disinfectant samples, the produced current signal was recorded by CA test (Figure S8), through which the H_2_O_2_ concentration was detected and calculated according to a regression equation obtained from the linear relationship in Figure 3d. The results are listed in Table 2, and the calculated recovery ratios of R-CeO_2_-O in medical disinfectants range from 98.16% to 101.95%, demonstrating its good detection accuracy towards H_2_O_2_ in real biological samples and its potential for large-scale applications.

## 4. Conclusions

In summary, CeO_2_ nanocrystals with selectively exposed facets were prepared by hydrothermal method and surface-modified with OVs through aluminothermic reduction. The synergistic effect of OVs and facets was investigated by the comparative analysis of the H_2_O_2_ non-enzymatic electrochemical sensing performance. Among all samples, R-CeO_2_-O exhibited the best sensing performance towards H_2_O_2_, which should give the credit to two reasons. Firstly, the (111) facet of CeO_2_ favors the generation of sub-surface OVs, which are more stable in the conventional environment and less prone to being re-occupied by oxygen-containing species. Secondly, linear OVs represent the dominant percentage on the (111) facet and generates highly identical electronic structures on the surface of R-CeO_2_-O, thus, unifying the proper bias voltage for a catalytic reaction, amplifying the sensing signal, and elevating the sensitivity. Moreover, the working mechanism of OVs on the (111) facet of CeO_2_ was investigated, and proved that OVs are beneficial in accelerating electronic transmission and producing new active sites, thus, facilitating the catalytic sensing reaction towards H_2_O_2_ molecules. Our investigation of the synergistic effect of OVs and facets provides necessary experimental and theoretical foundation for the future design of convenient, low-cost, and high-performance catalysts.

## Figures and Tables

**Table 1:** Comparison of sensitivity, linear range, and detection limit of various H_2_O_2_ sensors constructed on the basis of CeO_2_-dominated electrode nanomaterials.

	Sensitivity (μA mM^−1^ cm^−2^)	Linear Range (μM)	Detection Limit (μM)	References
R-CeO_2_-O	128.83	20–13,610	1.63	This work
CeO_2_-O	82.23	20–11,610	2.45	This work
^1^ CeO_2_/GC	-	91.88–2000	31.29	[48]
^2^ CeO_2_ NPs	0.126	50–1000	27	[49]
^3^ CeO_2_-HMTA	21.13	1–1000	0.6	[50]
		1000–25,000		
CeO_2_-fructose	9.6	10–1000	2	[50]
		1000–25,000		
Au/CeO_2_ NPs	1.150	4–2000	3	[49]
Au/CeO_2_	27.1	0–30	5	[51]
Pt/C-CeO_2_	185.4	10–3000	2	[52]
^4^ GS@CeO_2_/Au on GE	94.42	1–10,000	0.26	[38]
^5^ CeO_2_ NP/N-rGO	5.092	1.8–920.8	1.3	[53]

^1^ GC: glassy carbon. ^2^ NPs: nanoparticles. ^3^ HMTA: hexamethylene-tetra-amine. ^4^ GS: graphene sheets, GE: gold electrode. ^5^ N-rGO: nitrogen-doped reduced graphene oxide.

**Table 2:** Analytical results for the determination of H_2_O_2_ in medical disinfectant samples.

Added (μM)	Detected (μM)	RSD (%, n = 3)	Recovery (%)
80	81.560	2.83	101.95
160	157.08	1.73	98.160
300	301.96	2.20	100.65

## Data Availability

Not applicable.

## Supplementary Materials

The following supporting information can be downloaded at: https://www.mdpi.com/article/10.3390/bios12080592/s1, Figure S1: SEM and TEM images of R-CeO_2_-O; Figure S2: Electron paramagnetic resonance (EPR) spectra of CeO_2_-O and R-CeO_2_-O; Figure S3: CA curves of R-CeO_2_-O by continuously adding 1 mM H_2_O_2_ into 0.1 M PBS per 50 s at the bias voltage of −0.3 V, −0.4 V, −0.5 V and −0.6 V; Figure S4: SEM images of CeO_2_ catalysts with different morphologies: (a) CeO_2_-C (cube), (b) CeO_2_-R (rod) and (d) CeO_2_-S (sphere). (d) XRD patterns of CeO_2_ catalysts with different morphologies; Figure S5: (a) N_2_ adsorption-desorption isotherms and (b) Raman spectra of CeO_2_ nanocatalysts; Figure S6: Cyclic voltammetry curves of (a) CeO_2_-O and (b) R-CeO_2_-O; Figure S7: Amperometric response of R-CeO_2_-O upon the successive addition of 1 mM H_2_O_2_, Fe(NO_3_)_3_, Mn(NO_3_)_2_, Ca(NO_3_)_2_, Mg(NO_3_)_2_, AA, DA, and H_2_O_2_ in 0.1 M PBS at a bias voltage of −0.5 V; Figure S8: Amperometric response of R-CeO_2_-O after continuous addition of H_2_O_2_ solution to medical disinfectant with a bias of −0.5 V.
