# Supplementary material for: Oxygen Vacancy Injection on (111) CeO2 Nanocrystal Facets for Efficient H2O2 Detection
Source: Biosensors (Basel). 2022 Aug 3;12(8):592. doi: 10.3390/bios12080592 (PMC9405991; doi:10.3390/bios12080592)
Supplement: Supplementary file 1 [file biosensors-12-00592-s001.zip › biosensors-1830355-supplementary.pdf]

Supplementary Materials

Oxygen Vacancy Injection on (111) CeO_2 Nanocrystal Facets for Efficient H_2O_2 Detection

Tong Li †, Qi Wang † and Zhou Wang *

Key Laboratory of Liquid-Solid Structural Evolution and Processing of Materials of Ministry of Education, School of Materials Science and Engineering, Shandong University, Jinan 250061, China; litong1222@hotmail.com (T. L.); wangqi1016@sdu.edu.cn (Q. W.)
*Correspondence: wangzhou@sdu.edu.cn
†These authors contributed equally to this work.

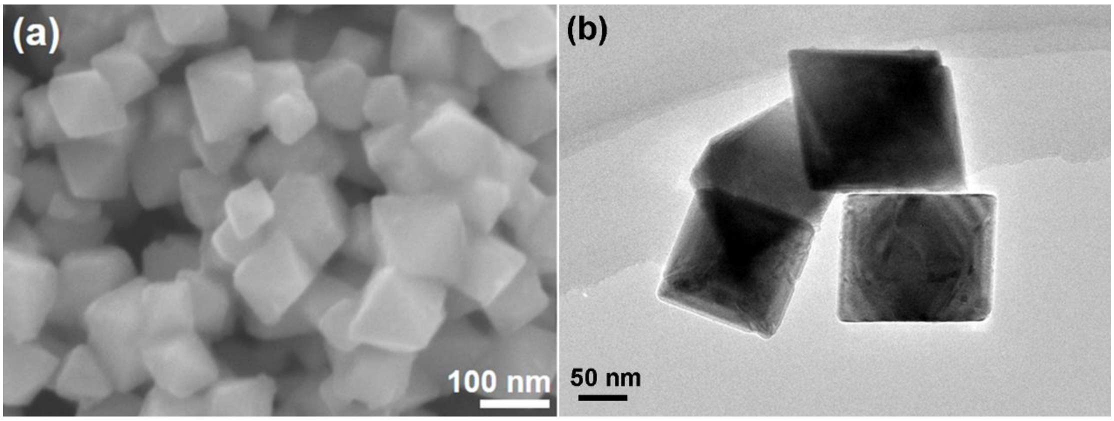

Figure S1. SEM and TEM images of R-CeO_2-O.

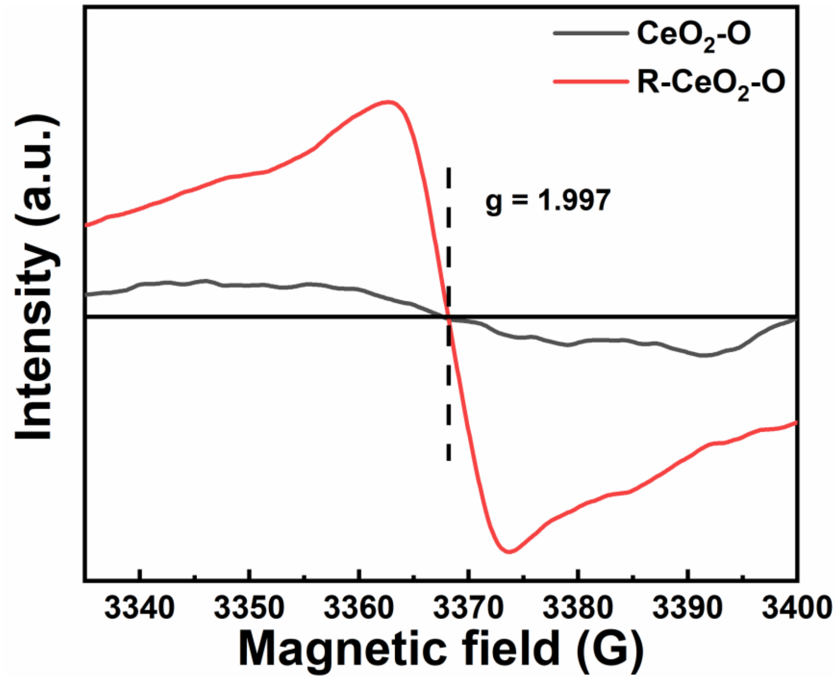

Figure S2. Electron paramagnetic resonance (EPR) spectra of CeO_2-O and R-CeO_2-O.

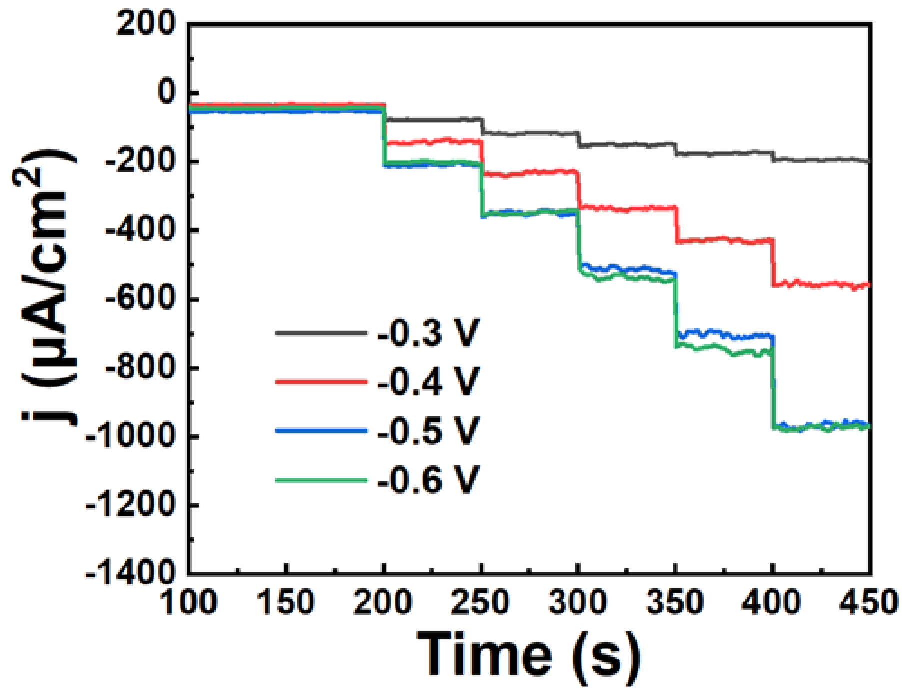

Figure S3. CA curves of R-CeO_2-O by continuously adding 1 mM H_2O_2 into 0.1 M PBS per 50 s at the bias voltage of −0.3 V, −0.4 V, −0.5 V and −0.6 V.

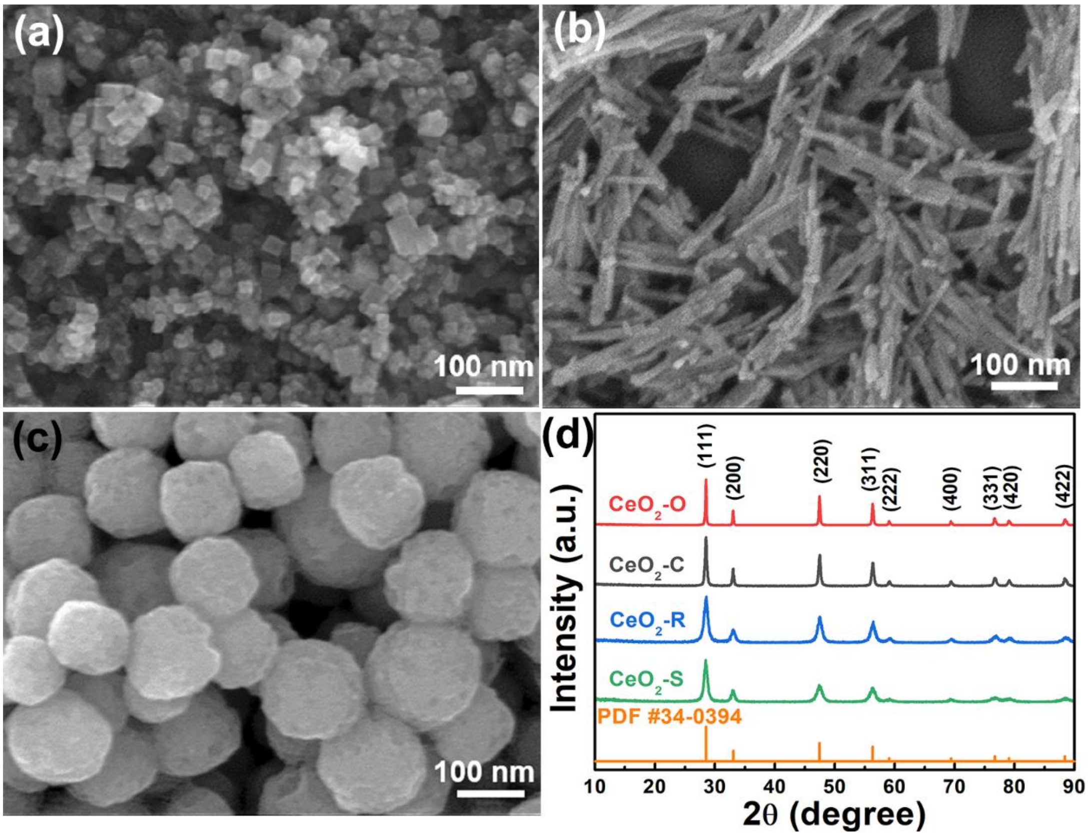

Figure S4. SEM images of CeO_2 catalysts with different morphologies: (a) CeO_2-C (cube), (b) CeO_2-R (rod) and (d) CeO_2-S (sphere). (d) XRD patterns of CeO_2 catalysts with different morphologies.

Figure S4a exhibits well-developed inhomogeneous cubes (CeO_2-C) with side length ranging from 10 nm to 40 nm. In Figure S4b, the diameter of the crisscross CeO_2-R is about 10 nm with a length of about 150 nm. Figure S4c shows uniform CeO_2-S with the diameter of 150 nm. Phase uniformity must be strictly disciplined to eliminate interference from basic crystallographic structure. The phase composition of CeO_2 catalysts with different morphologies was investigated by XRD. As shown in Figure S4d, all samples show identical diffraction pattern and match the standard card with no extra peak, which proves the high purity and good phase consistency. Based on the above discussion, CeO_2 catalysts with diverse exposed facets were fabricated, which successfully completed the groundwork for further study.

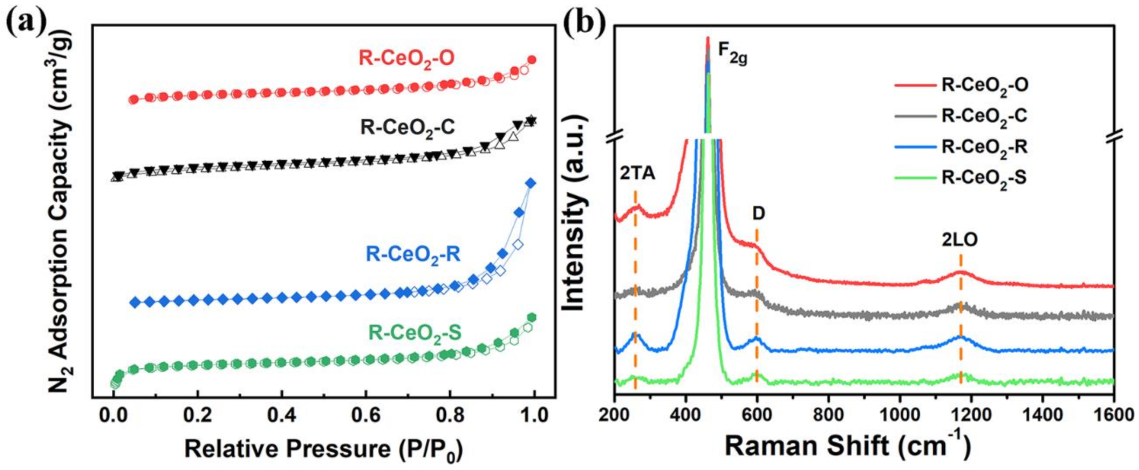

Figure S5. (a) N_2 adsorption-desorption isotherms and (b) Raman spectra of CeO_2 nanocatalysts.

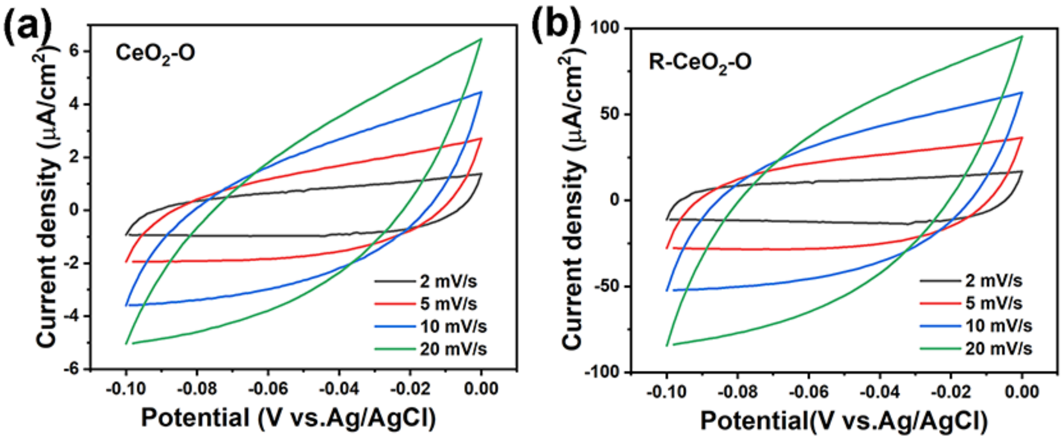

Figure S6. Cyclic voltammetry curves of (a) CeO_2-O and (b) R-CeO_2-O.

The EASA test is conducted on CeO_2-O and R-CeO_2-O electrode in N_2-saturated 0.1 M PBS. The CV curves of CeO_2-O and R-CeO_2-O electrodes at different scanning rates in the non-faradic regions (−0.1 V-0 V) are shown in Figure S6. By taking the anode current density at −0.02 V and the cathode current density at -0.08 V, the slope was obtained after linear fitting, then the following formula can be used to calculate the electric double layer specific capacitance (C_{dl}):

$$C_{dl} = \frac{(\text{Slope1} - \text{Slope2}) * 1000}{2} = 500 * (\text{Slope1} - \text{Slope2})$$

It could be calculated that the C_{dl} of CeO_2-O and R-CeO_2-O were 203.16 μF/cm^2 and 3.39 mF/cm^2, respectively. The electrochemical active surface area (EASA) can be calcuated through the following formula

$$EASA = C_{dl} / C_s$$

It was assumed that, the specific capacitance of flat standard surface (C_s) with 1 cm^2 of real surface which was assume the value around 40 μF/cm^2. The calculated values of the corresponding samples are 5.08 cm^2 (CeO_2-O) and 84.75 cm^2 (R-CeO_2-O).

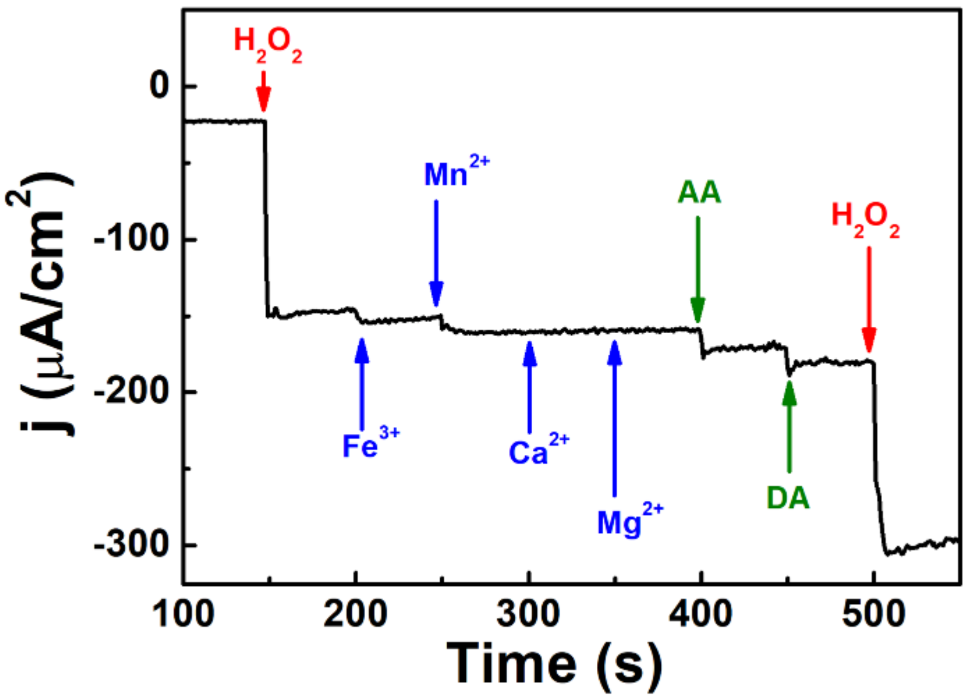

Figure S7. Amperometric response of R-CeO_2-O upon the successive addition of 1 mM H_2O_2, $Fe(NO_3)_3$, $Mn(NO_3)_2$, $Ca(NO_3)_2$, $Mg(NO_3)_2$, AA, DA and H_2O_2 in 0.1 M PBS at a bias voltage of -0.5 V.

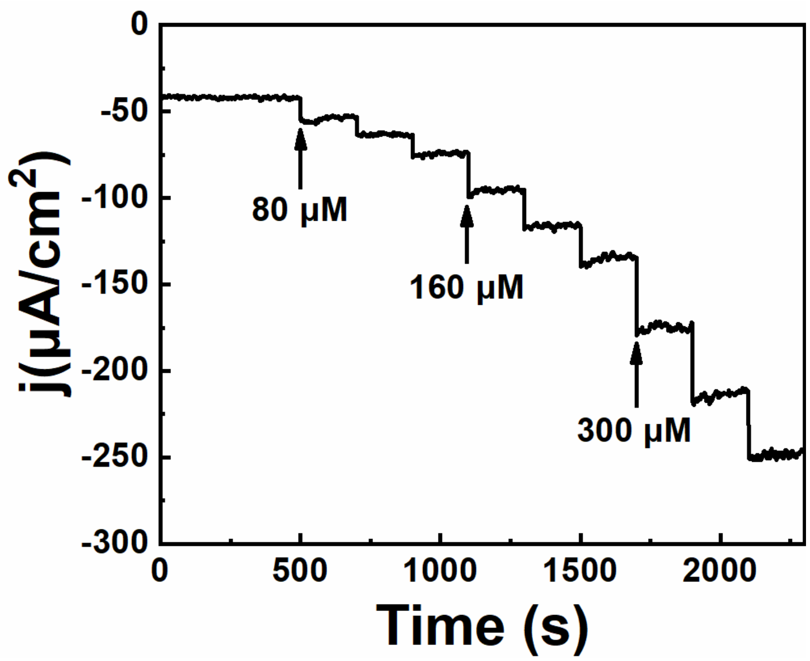

Figure S8. Amperometric response of R-CeO_2-O after continuous addition of H_2O_2 solution to medical disinfectant with a bias of −0.5 V.
